# Constructing a Model Using Clock‐Related lncRNAs for Predicting the Tumor Microenvironment of Gliomas

**DOI:** 10.1002/brb3.71000

**Published:** 2025-10-15

**Authors:** Mingjie Gong, Chengfa Sun, Zhenhua Shi, Junxiang Wang, Weiwei Zhai, Zhengquan Yu

**Affiliations:** ^1^ Department of Neurosurgery The First Affiliated Hospital of Soochow University Suzhou Jiangsu Province China; ^2^ Department of Neurosurgery Changshu No.2 People's Hospital, Affiliated Changshu Hospital of Nantong University Changshu Jiangsu Province China

## Abstract

**Purpose:**

Circadian locomotor output cycles kaput (CLOCK) and its related genes play important roles in cellular functions. This study aims to construct a predictive model for CLOCK‐related genes and identify lncRNAs that may influence Tumor Microenvironment of Glioma.

**Method:**

We included bulk RNA‐sequencing data and clinical information for glioma samples from the TCGA and CGGA databases. Univariate Cox and LASSO–Cox analyses were used to screen CLOCK‐related genes. Consensus clustering was applied to classify glioma samples, followed by differential gene expression analysis. CLOCK‐related lncRNAs were identified through correlation analyses, hub lncRNAs were selected using LASSO–Cox, and their expression was validated by qPCR in cultured glioma cell lines.

**Finding:**

We identified nine CLOCK‐related genes, and unsupervised clustering based on these genes divided glioma samples into three clusters. Enrichment analysis revealed that genes differentially expressed between the high CLOCK‐related cluster and other clusters were enriched in immune‐related molecular functions. Co‐expression analysis detected 102 potentially correlated lncRNAs. We constructed a CLOCK‐related lncRNA risk score based on 31 of these lncRNAs. Subsequent multivariable Cox analysis identified 9 hub lncRNAs, and accuracy testing demonstrated the model's good performance. Immune infiltration analysis showed higher stromal, immune, and ESTIMATE scores in the high CLOCK‐related lncRNA score group.

**Conclusion:**

CLOCK‐related RNAs and lncRNAs play distinct roles within the glioma microenvironment. These findings offer new insights into the challenges that need to be addressed when using immunotherapeutic approaches to treat gliomas.

## Graphical abstract

Step 1: Identify hub clock‐related genes through differential expression analysis, univariate Cox analysis, and LASSO‐Cox regression; Step 2: Identify and screen hub clock‐related lncRNAs using correlation analysis and LASSO‐Cox regression; Step 3: Validate the expression differences of the selected lncRNAs in cell experiments using qPCR.

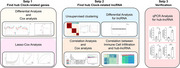

## 1 Background

Gliomas are malignant primary tumors that show high levels of invasiveness and mortality. They advance quickly, and are associated with a poor prognosis and limited patient survival (Zhang and Zhang 2020, Tan et al. 2020). The reported annual incidence of gliomas is 0.0064%, and the highest rate of glioblastoma (GBM) is 0.00403% (Ostrom et al. 2022). Clinical studies have shown that the median overall survival for elderly patients with glioma is only 10.5 months (Bruno et al. 2022). Patients with glioma are usually managed through surgical procedures accompanied by chemotherapy with alkylating agents such as temozolomide, tumor‐treating field therapy, and immunotargeted therapy (Molinaro et al. 2020, Breen et al. 2020, Khan et al. 2016, Herrlinger et al. 2019, Lombardi et al. 2019, Weller et al. 2015).

Immunotherapy uses antibodies and tumor‐specific receptors to eliminate tumors (Lesch and Gill 2021), and its use in solid tumors is becoming increasingly common (Sampson et al. 2020). Immunotherapy can prolong the life expectancy of patients and enhance their quality of life (Lesch and Gill 2021). However, awareness regarding immune therapy resistance or the innate features of GBM in tumors is currently lacking (Ettinger et al. 2022). The primary impediments for glioma immunotherapy are the strong inhibitory effects of the tumor immune environment and immune resistance. The response rate to immunotherapy is restricted, and anticipating the clinical efficacy and potential side effects is challenging (Zhang and Zhang 2020). Consequently, identification of novel immunotherapeutic targets is essential to enhance the prognosis of patients with glioma.

The biological clock gene is one of the elements that regulate the immune system (Curtis et al. 2014, Scheiermann et al. 2013, Zhang et al. 2021). In studies analyzing the effects of the light/dark cycle, the phagocytic activity of peritoneal macrophages collected from mice during the later stages of light exposure was found to be more forceful than that during other periods of the day (Hayashi et al. 2007). Blood samples taken from humans have shown that T‐cell numbers tend to be higher at night, lower in the morning, and remain relatively low throughout the day (Besedovsky et al. 2014).

Within cells, there exists a circadian rhythm regulator known as circadian locomotor output cycles kaput (CLOCK), which can cooperate with BMAL1 and other factors to regulate the expression of downstream genes, thereby affecting cellular self‐renewal and metabolism—this is referred to as the “CLOCK phenomenon” (Chen et al. 2020). Studies have demonstrated that components of the circadian clock can facilitate the infiltration of small tumor cells into gliomas, which, in turn, can suppress the immune system, indicating that the circadian clock influences tumor immune infiltration (Xuan et al. 2022). Immune cells play vital roles in identifying, eliminating, or maintaining tumors, and the expression of circadian clock genes can influence tumor immunity and cell growth (Wang et al. 2021).

Numerous studies on cancer have revealed that long noncoding RNAs (lncRNAs) influence the expression of genes related to cell cycle, survival, and metastasis (Taniue and Akimitsu 2021). Moreover, the regulatory roles of lncRNAs in energy metabolism and the development of tumor resistance in cancer have further confirmed the close relationship between lncRNAs and the development and occurrence of tumors (Lin et al. 2020). lncRNAs show a circadian rhythm and also influence the circadian rhythm by controlling clock genes (Cheng et al. 2007, Cui et al. 2015). The precise mechanisms underlying lncRNA‐mediated regulation of circadian rhythms remain unclear, although some researchers have posited that they are similar to those of microRNA (miRNA) (Quinn and Chang 2016).

In gliomas, certain molecular features are significantly associated with patient prognosis (Saaid et al. 2022), making it necessary to explore additional prognostic molecular markers. Circadian clock genes have been hypothesized to be closely linked to the survival outcomes of patients with gliomas during immunotherapy. Enhancing our understanding of the connection between circadian rhythms and the immune cycle in gliomas could lead to the development of successful immunotherapy regimens. Using prognostic machine‐learning approaches, we identified glioma‐relevant CLOCK‐related genes and, via additional analytical frameworks, prioritized candidate lncRNAs, thus extending current knowledge of CLOCK‐mediated biology in glioma.

## 2 Methods

### 2.1 Acquisition of the CLOCK‐Related Gene Set

The CLOCK‐related gene set used in this study was sourced from previously published reviews and original studies on the CLOCK phenomenon. A literature search was conducted using the search terms (CLOCK and [Glioma or glioblastoma or low‐grade gliomas or GBM or LGG] and [mRNA or GENE OR]).

### 2.2 Data Collection

The glioma patient transcriptome profiles (normalized fragments per kilobase million [FPKM] and counts) and corresponding clinical and pathological data included in the study were sourced from The Cancer Genome Atlas (TCGA) and Chinese Glioma Genome Atlas (CGGA) databases. Count‐normalized transcriptome profiles were used for differential analysis, whereas FPKM‐normalized transcriptome profiles were used for other analyses. The transcriptome profiles of normal brain tissues (count‐normalized) were obtained from the Genotype‐Tissue Expression (GTEx) project, a resource that provides mRNA sequencing (mRNA‐seq) data for various human tissues and organs. No further processing was applied to the sample data included in this study, except for logarithmic transformation and z‐score calculation used for visualization purposes. For different datasets, we treated each as an independent analysis cohort and did not apply batch‐effect correction. This strategy helps avoid masking the genuine biological effects of individual genes. We then integrated results across cohorts to minimize potential methodological bias between datasets.

### 2.3 Cell Culture

Human astrocyte cell line (HA) and human glioblastoma cell line U87 were obtained from the Shanghai Institute of Biochemistry and Cell Biology. The cells were cultured in Dulbecco's Modified Eagle Medium (DMEM) supplemented with 10% fetal bovine serum (FBS; obtained from Shanghai Zhongqiao Xinzhou Biotechnology Co., Ltd). All cells were incubated in a humidified atmosphere with 5% CO_2_ at 37°C.

### 2.4 Signature Analysis by Quantitative Real‐Time Polymerase Chain Reaction

Total RNA from the aforementioned cell lines was extracted using NcmZol Reagent (New Cell & Molecular Biotech Co., Ltd., M5100). Complementary DNA (cDNA) was synthesized with HiScript III All‐in‐one RT SuperMix Perfect for quantitative polymerase chain reaction (qPCR; R333; Vazyme, China). qPCR was performed using TB Green Premix Ex Taq II (Tli RNaseH Plus; RR820A; Takara) on an Applied Biosystems StepOnePlus Real‐Time PCR System (Thermo Fisher Scientific). The 2^−ΔΔ^Ct method was used to normalize the results. The primers used in this study were sourced from RiboBio (Guangzhou, China), and their sequences are listed in Supplementary Table 1.

### 2.5 Differential Expression Analysis of mRNA and lncRNA

In this study, we conducted differential analysis of mRNA expression between the two groups using the R language limma package. We used the R packages ggplot2 and ggsignif for boxplot visualization. Volcano plots were generated to filter out differential results using the criteria of *p* < 0.001 and log fold change (logFC) > 2. For visualization, we employed the R packages ggpubr and ggtheme.

### 2.6 Construction and Verification of the Survival Model

We collected expression profiles and clinical information for CLOCK‐related genes and associated lncRNAs from glioma samples in the TCGA and CGGA databases. Initially, we performed univariate Cox analysis of the expression of these genes. Genes with *p*‐values less than 0.01 in the univariate Cox regression analysis from both TCGA and CGGA databases were selected, and the genes that appeared in both cohorts were designated as the CLOCK‐hub genes. Subsequently, we employed the R package (glmnet) to implement the least absolute shrinkage and selection operator (LASSO)‐Cox machine learning algorithm to construct the CLOCK risk score. The risk score was calculated as follows:

RiskScore=∑ni=1(Coef∗xi)

In the equation, “n” represents the sample size, “coef” is the regression coefficient from the multivariate Cox analysis for a single mRNA, and “Xi” denotes the expression level of a single mRNA. All samples were divided into high‐ and low‐risk groups on the basis of the median risk scores. Subsequently, time‐dependent receiver operating characteristic curves (ROC), calibration curves, and decision curve analysis plots were generated to validate the accuracy of the risk score. To visualize the accuracy of the risk score, we used the nomogram analysis function of the online analysis platform Sangerbox (www.sangerbox.com). This involved incorporating the risk score, isocitrate dehydrogenase (IDH) mutation status, WHO grading, and 1p/19q chromosomal deletion status of each sample to create a nomogram plot.

### 2.7 Consensus Clustering

Consensus clustering was performed on glioma samples from the TCGA using the Sangerbox online analysis platform. Clustering was based on the expression matrix of the CLOCK‐hub gene. The number of clusters was selected by considering the area under the cumulative distribution function (CDF) curve, the trend of CDF delta descent, and the average within‐cluster consistency. Clusters highly related to CLOCK were identified for subsequent analyses.

### 2.8 Functional Enrichment Analysis of Differentially Expressed Genes

To explore the functional differences among the different clusters, we identified differentially expressed genes between the high CLOCK‐related cluster and the other two clusters. We obtained GSEA software (version 3.0) from the GSEA website (http://software.broadinstitute.org/gsea/index.jsp) and downloaded the c5.go.mf.v7.4. symbols.gmt subset from the Molecular Signatures Database (https://doi.org/10.1093/bioinformatics/btr260, http://www.gsea‐msigdb.org/gsea/downloads.jsp) to assess the relevant pathways and molecular mechanisms.

### 2.9 Selection of CLOCK‐Related lncRNAs

Through correlation analysis, we examined the correlation between clock‐related genes and differentially expressed lncRNAs in clusters 1, 2, and 3. We identified the top 20 lncRNAs that correlated with each gene, evaluated the intersection, and found lncRNAs simultaneously associated with two or more clock‐related genes.

### 2.10 Immune Cell Infiltration in the Tumor Microenvironment

We assessed the immune cell infiltration levels of glioma samples from the TCGA using the TIMER and Es algorithms. The TIMER algorithm was used to calculate the infiltration levels of CD4 + T cells, CD8 + T cells, B cells, macrophages, neutrophils, and dendritic cells (DCs) in the samples. The Es algorithm computed the stromal and immune scores of the samples. Visualization was performed using heatmaps, correlation plots, and violin plots. On the basis of the CLOCK‐related lncRNA risk scores, samples were categorized into high‐ and low‐risk groups, and the differences in immune infiltration between the two groups were examined.

## 3 Results

### 3.1 Expression of CLOCK‐Related Genes in Gliomas and Their Correlation With Survival

**FIGURE 1:**
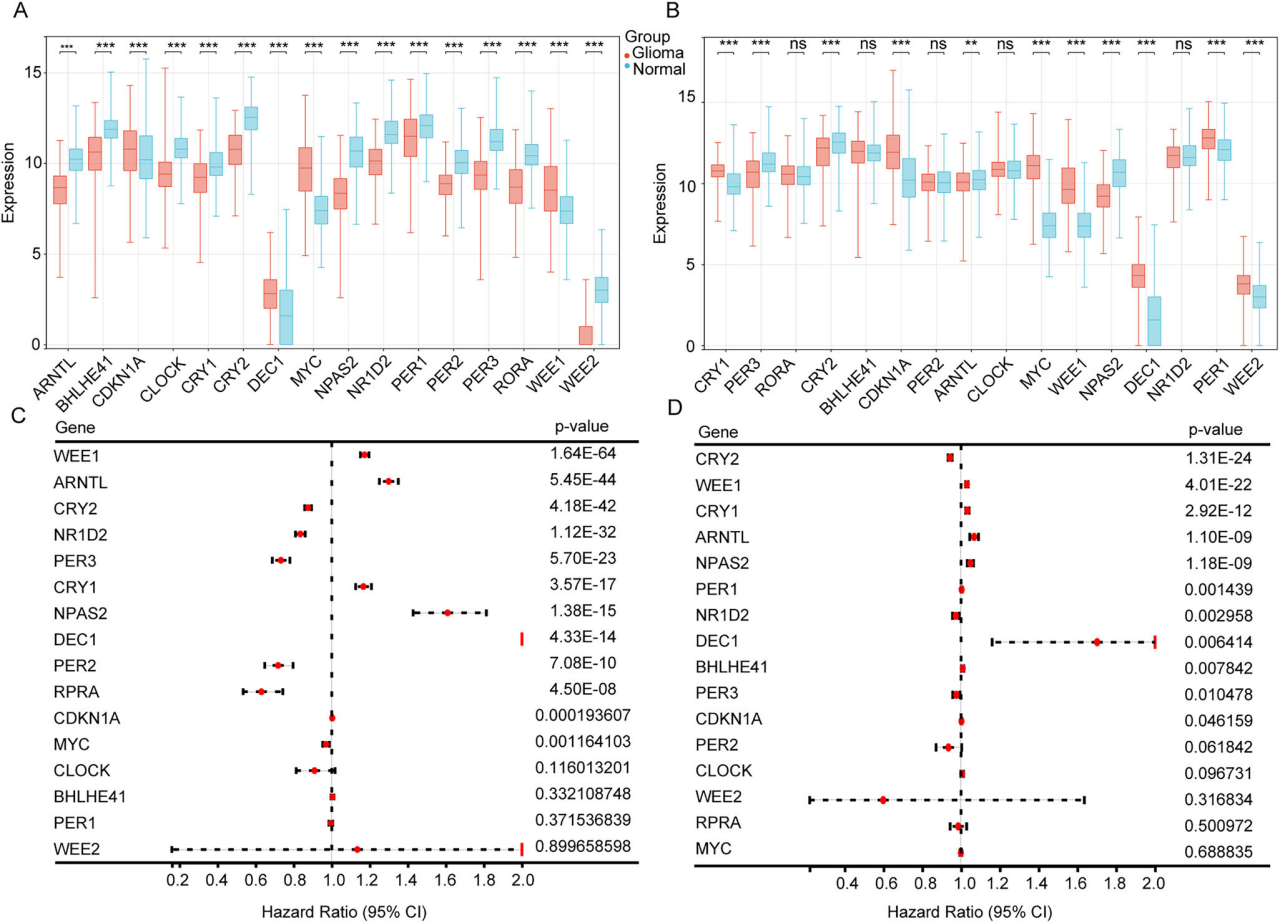
Expression of CLOCK‐related genes and their correlation with survival in different databases. A, B: differential expression of CLOCK‐related genes between glioma samples and normal tissues from online databases; (A) TCGA compared to GTEx and (B) CGGA compared to GTEx. (C, D) single‐factor Cox analysis incorporating patient survival information: (C) TCGA and (D) CGGA. **Abbreviations**: CGGA, Chinese Glioma Genome Atlas; GTEx, genotype‐tissue expression; TCGA, The Cancer Genome Atlas. *Note*: *p*‐values were calculated from log‐rank tests, and *p* < 0.05 was considered statistically significant.

After an extensive literature review, we identified 16 CLOCK‐related genes (Wang and Chen 2022, Sancar and Van Gelder 2021, Kinouchi and Sassone‐Corsi 2020). We observed diverse expression patterns of CLOCK‐related genes by comparing samples from the TCGA and CGGA databases with those from the GTEx database. Some genes, such as ARNTL (BMAL1), CRY2, NPAS2, and PER3, exhibited significantly lower expression in glioma samples from both the TCGA and CGGA databases (*p* < 0.05). In contrast, WEE1, MYC, DEC1, and CDKN1A showed elevated expression in tumors (*p* < 0.05; Figures 1A,B).

Univariate survival analysis revealed that nine of the 16 CLOCK‐related genes showed significant associations with prognosis in both TCGA and CGGA samples (Figure 1C,D). Among them, CRY2, NR1D2, and PER3 were negatively correlated with survival, indicating that higher expression of these genes is associated with shorter survival. Typically, lower gene expression in tumors is correlated with better prognosis. However, our results indicate a different trend, suggesting that this phenomenon may be related to distinct nodes of the biological clock within the tumor.

### 3.2 Survival Prognostic Model Based on CLOCK‐Related Genes

**FIGURE 2:**
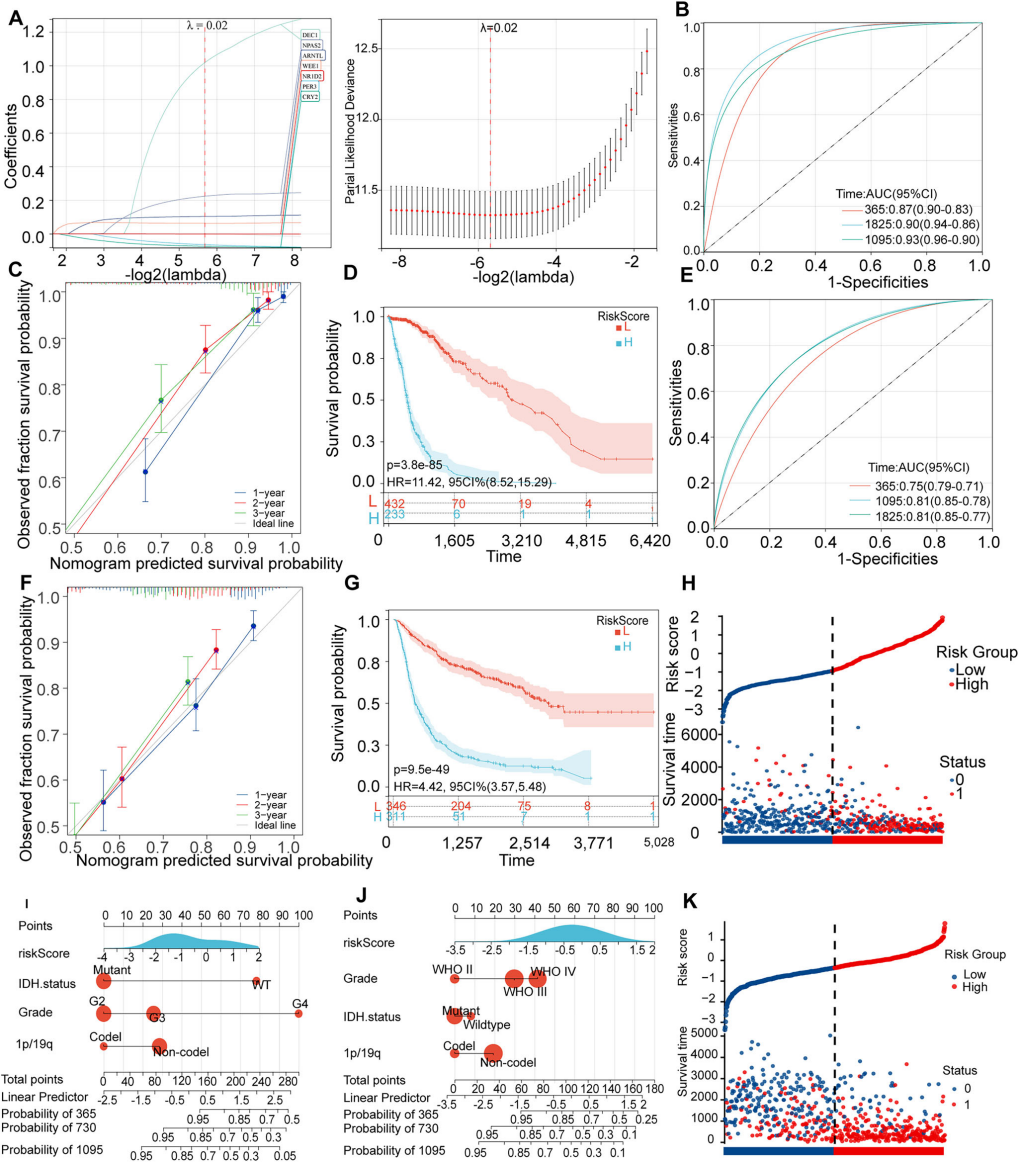
Construction and validation of the CLOCK‐related risk score. (A) Log(lambda) sequence plot of CLOCK‐related genes obtained through LASSO regression and the distribution of LASSO coefficients for CLOCK‐related genes in the TCGA dataset; (B) time‐dependent ROC analysis of the CLOCK‐related risk score in TCGA; (C) calibration plots predicting 1‐, 2‐, and 3‐year survival in the TCGA samples; (D) survival analysis of the high and low‐risk score groups in the TCGA samples; (E) time‐dependent ROC analysis of the CLOCK‐related risk score in the CGGA samples; (F) calibration plots predicting 1‐, 2‐, and 3‐year survival in the CGGA samples; (G) survival analysis of the high and low‐risk score groups in the CGGA samples; (H, K) prognostic risk models based on CLOCK‐related risk scores in the TCGA and CGGA samples; and (I, J) nomogram with CLOCK‐related risk score groups for predicting the overall survival of glioma patients, constructed on the basis of the TCGA and CGGA datasets.

To comprehensively analyze the relationship between CLOCK‐related genes and survival, we included nine genes that showed significant prognostic correlations in glioma samples from both the TCGA and CGGA databases. Using the LASSO‐Cox method, we constructed a survival prognosis model for samples from the TCGA database. Subsequently, CLOCK‐related risk scores were assigned to each sample (Figure 2A). Time‐dependent area under the curve (AUC) analysis indicated high accuracy of the model in predicting survival times for glioma samples (95% CI of the AUC: one year, 0.90–0.83; two years, 0.96–0.90; and three years, 0.94–0.86; Figure 2B). Calibration curves demonstrated satisfactory accuracy for survival prediction at one, two, and three years (Figure 2C). Survival differences between the groups with high and low CLOCK‐related risk scores revealed that patients with higher CLOCK‐related risk scores had a poorer prognosis (Figure 2D). Moreover, by the end of the TCGA follow‐up period, the high‐risk group showed more cases of deaths (Figure 2H).

A nomogram was constructed to visualize the relationship between CLOCK‐related risk scores and prognosis by incorporating other critical clinical factors in gliomas. The results showed that CLOCK‐related risk scores exhibited a good distribution pattern within the model (Figure 2J).

In the CGGA samples, we similarly constructed CLOCK‐related risk scores based on the nine identified genes, and the results demonstrated a predictive trend consistent with that observed in TCGA samples. On the basis of these findings, we considered CLOCK‐related risk scores to be essential independent factors affecting the survival time of patients with glioma (Figure 2E–G,J,K).

### 3.3 The Expression Patterns of CLOCK‐Related Genes Were Different in Glioma Samples

**FIGURE 3:**
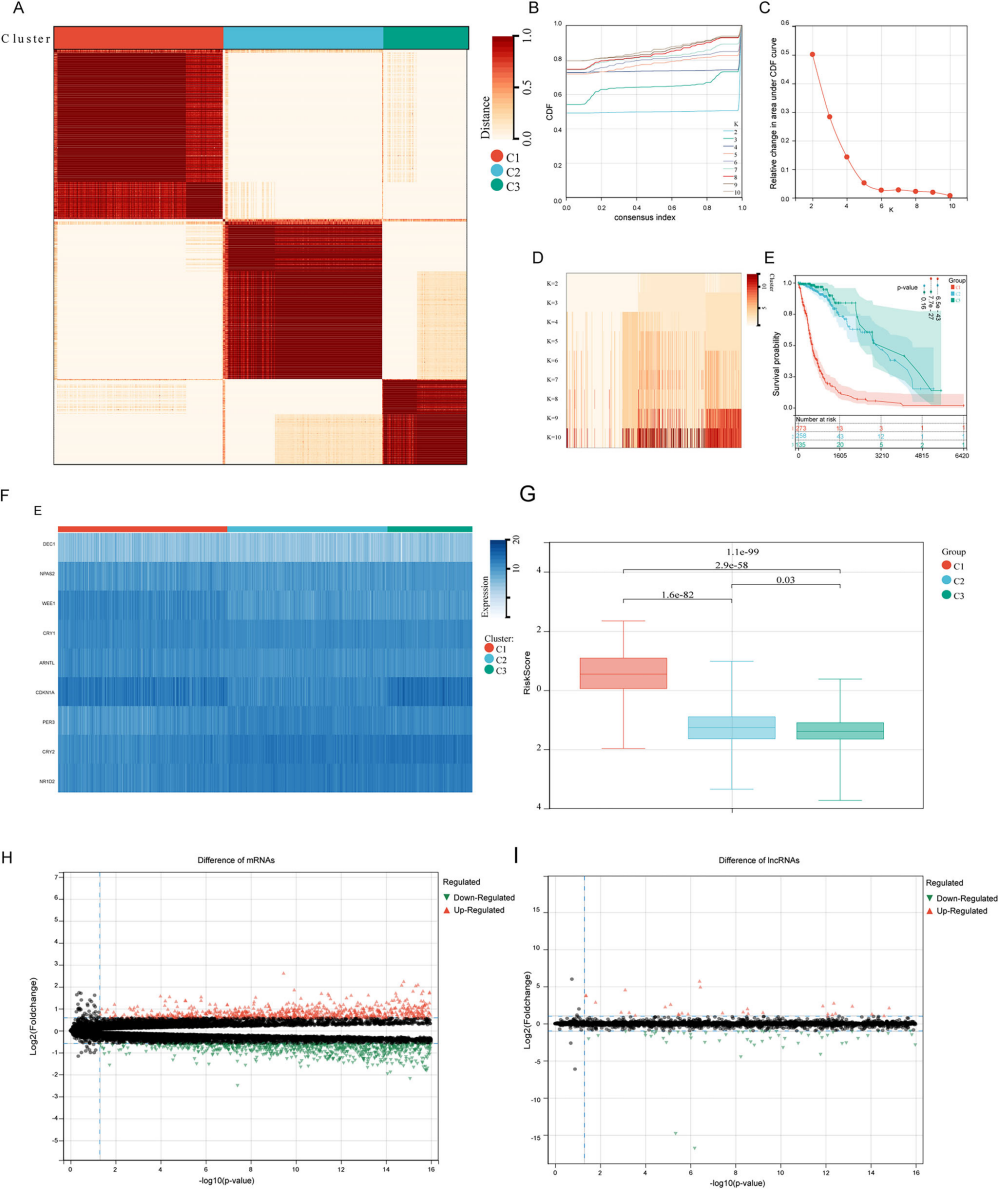
Identifying the high CLOCK‐related cluster in TCGA samples and analyzing inter‐cluster differences. (A) Unsupervised clustering method applied to the transcriptional profiles of 673 glioma samples from TCGA; (B–D) determination of cluster number (k): (B) cumulative distribution function, (C) downward trend in CDF delta, and (D) average consistency within the clustering groups; (E) differences in survival time among the three clusters; (F) heatmap depicting the expression of CLOCK‐related genes among the three clusters; (G) differences in CLOCK‐related risk scores among the three clusters; (H, I) volcano plots depicting differential genes between the high CLOCK‐related cluster and other clusters: (H) mRNA and (I) lncRNA. *Note*: *p*‐values were calculated from log‐rank tests, and *p* < 0.05 was considered statistically significant.

To explore tumor‐specific differences in the occurrence of the CLOCK phenomenon in glioma samples, we analyzed the expression patterns of these nine CLOCK‐related hub genes and constructed an unsupervised consensus clustering model (Figure 3A). Considering the CDF area (Figure 3B), the CDF delta downward trend (Figure 3C), and the average consistency within the clustering groups (Figure 3D), we selected K = 3 and divided the samples into three clusters. Kaplan–Meier survival curves of the three clusters revealed significant differences in survival times, with Cluster 1 showing a worse prognosis (*p* = 6.5e‐43) (Figure 3E).

Analyzing the expression of CLOCK‐related genes among the three clusters, we defined Cluster 1 as the high CLOCK phenomenon cluster since it showed high expression of genes such as ARNTL (BMAL1) and WEE1. Clusters 2 and 3 were defined as the low CLOCK phenomenon clusters (Figure 3F). CLOCK‐related risk scores also showed significant differences among the three clusters, with Cluster 1 containing samples with higher CLOCK‐related risk scores (*p* = 1.1e‐99) (Figure 3G).

### 3.4 Highly Expressed Genes in the High CLOCK Phenomenon Cluster Were Associated With Immunity

**FIGURE 4:**
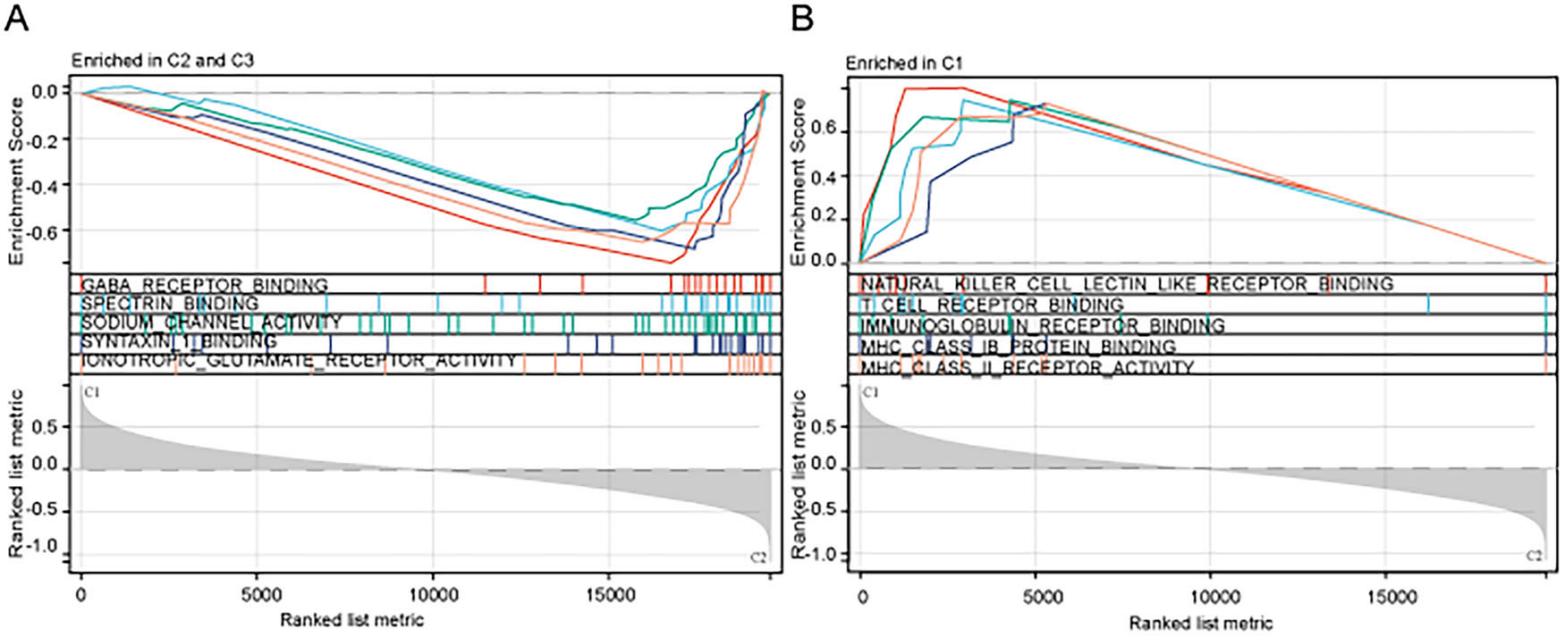
Molecular functional enrichment of differentially expressed genes between the high CLOCK‐related cluster and other clusters. (A) Enrichment of molecular functions in other clusters and (B) Enrichment of molecular functions in the high CLOCK‐related cluster.

We conducted a differential expression analysis of the entire genome between the high and low CLOCK phenomenon clusters and identified 2000 mRNAs and 422 lncRNAs with differential expression between the two clusters (Figure 3H–I, Supplementary Tables 1 and 2). Enrichment analysis of the differentially expressed mRNAs revealed several genes enriched in immune‐related pathways, suggesting a potential association between the CLOCK phenomenon and immune response in gliomas (Figure 4A,B).

### 3.5 Clock‐Related lncRNAs Can Also be Used as Independent Prognostic Factors

**FIGURE 5:**
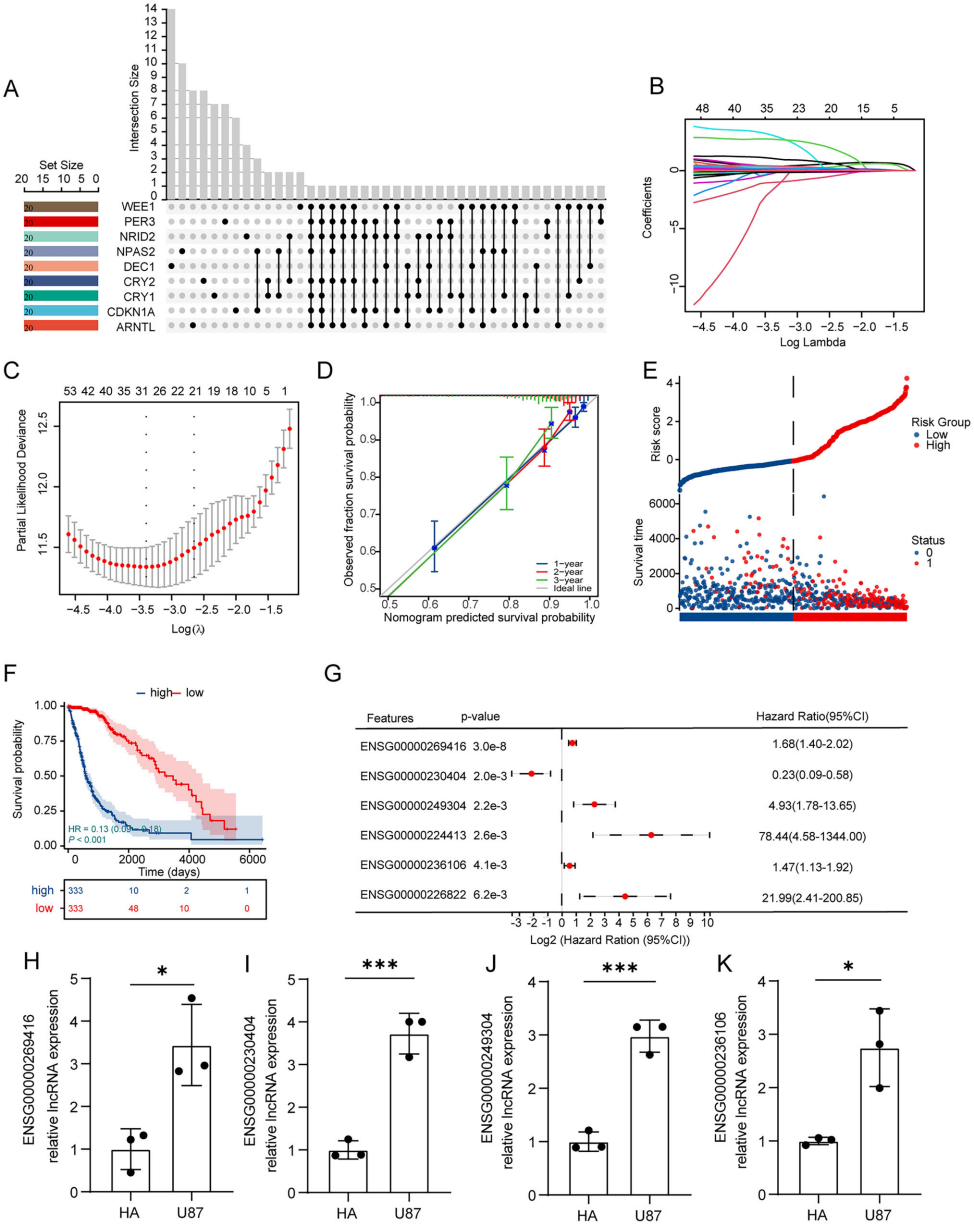
Filtering of CLOCK‐related lncRNAs and construction of a CLOCK‐related lncRNA risk score. (A) Visualization of intersection information of lncRNAs after correlation analysis. (B, C) Log(lambda) sequence plot of CLOCK‐related lncRNAs obtained through LASSO regression and the distribution of LASSO coefficients for CLOCK‐related lncRNAs in the TCGA dataset. (D) Calibration plots predicting 1‐, 2‐, and 3‐year survival in the TCGA sample. (E) Prognostic risk models based on CLOCK‐related lncRNA risk scores in the TCGA sample. (F) Analysis of survival differences between the high‐ and low‐risk groups. (G) Multifactor Cox selection of hub CLOCK‐related lncRNAs after LASSO analysis. (H–K): qPCR analysis was conducted to examine the expression levels of the four hub lncRNAs in U87 and HA cells. *Note*: *p*‐values were calculated from log‐rank tests, and *p* < 0.05 was considered statistically significant. *p < 0.05; **p < 0.01; ***p < 0.001.

By conducting correlation analysis of the 422 differentially expressed lncRNAs and CLOCK‐related genes identified in the two clusters, we selected the top 20 lncRNAs with the highest correlation for each gene. Considering the intersection of the lncRNAs related to each gene, we identified 102 lncRNAs associated with two or more CLOCK‐related genes (Figure 5A, Supplementary Table 3). Subsequently, LASSO‐Cox analysis of these 102 lncRNAs identified 31 lncRNAs that were used to construct a CLOCK‐related lncRNA risk score (Figure 5B, Supplementary Table 4). This model demonstrated good accuracy (Figure 5D–E), and grouping the high and low CLOCK‐related lncRNA risk scores by the median showed significant differences in survival (Figure 5F).

Multifactorial analysis of these 31 lncRNAs revealed that nine lncRNAs exhibited statistically significant differences (Supplementary Table 5). These results suggested that these nine lncRNAs may be accurate factors for predicting the survival of patients with glioma, and we refer to them as CLOCK‐related hub lncRNAs (Figure 5G). The qPCR results for the four lncRNAs showed that their expression levels were significantly higher in glioma cell lines than in astrocytes (Figure 5H–K).

### 3.6 The Expression of CLOCK‐Related Hub lncRNAs in Different CLOCK Phenomenon Clusters Is Different

**FIGURE 6:**
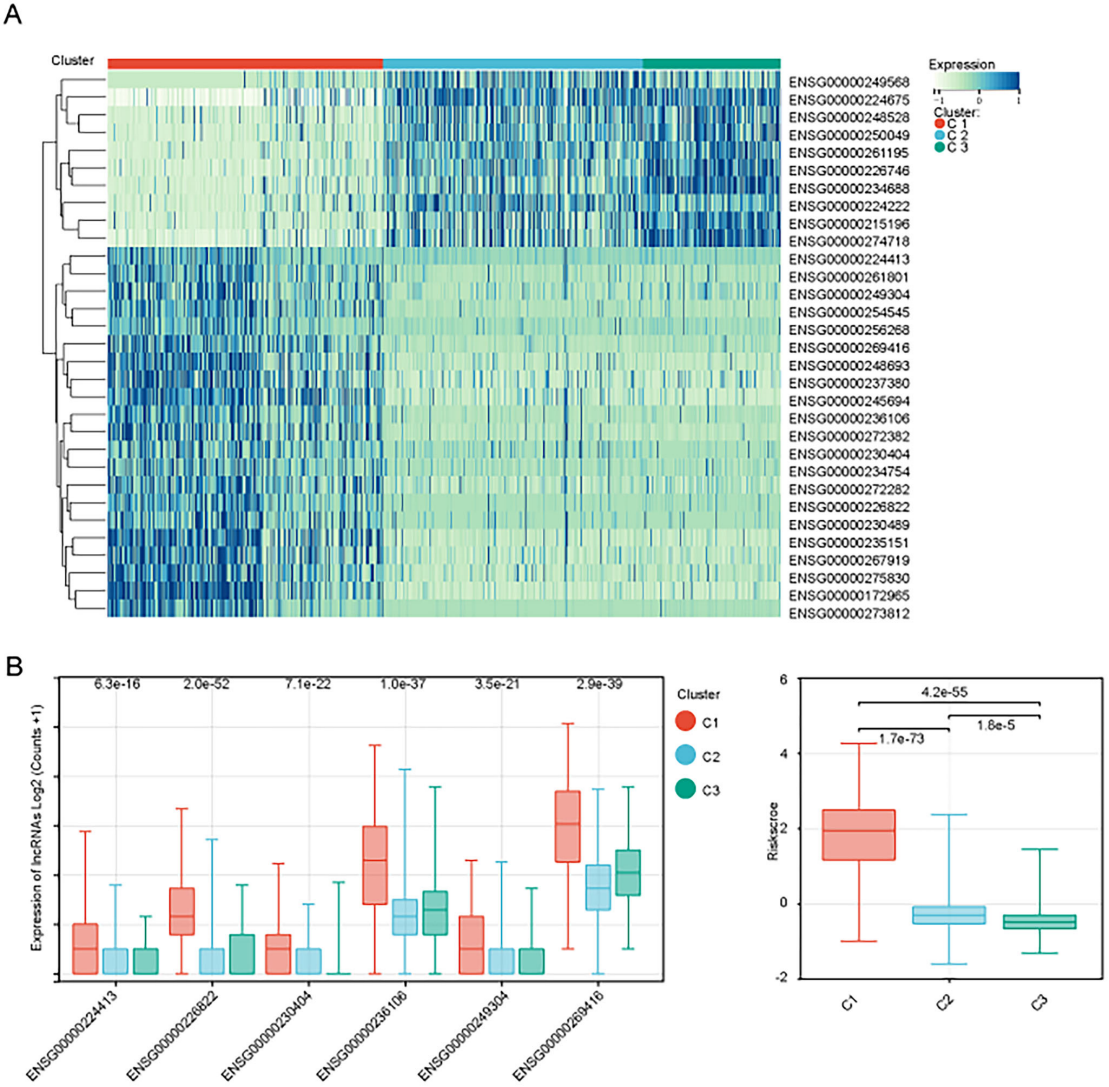
Expression Patterns of CLOCK‐Related lncRNAs among Different Clusters. (A) Heatmap depicting the expression of lncRNAs included in the LASSO model across different clusters, (B) expression profiles of CLOCK‐hub lncRNAs among the three clusters, and (C) distribution of CLOCK‐related lncRNA risk scores across the three clusters.

We visualized the expression of 31 lncRNAs screened using LASSO‐Cox in different clusters of the CLOCK phenomenon. The results showed that the expression patterns of these lncRNAs in clusters 1, 2, and 3 were significantly different (Figure 6A). Clock‐associated hub lncRNAs associated with worse prognosis were significantly overexpressed in the high CLOCK phenomenon cluster (Figure 6B). The clock‐associated lncRNA risk scores also differed among the three clusters. The samples in Cluster 1 had higher scores (Figure 6C).

### 3.7 High Clock‐Associated lncRNAs Risk Assessment Group Had Higher Immune Cell Infiltration

**FIGURE 7:**
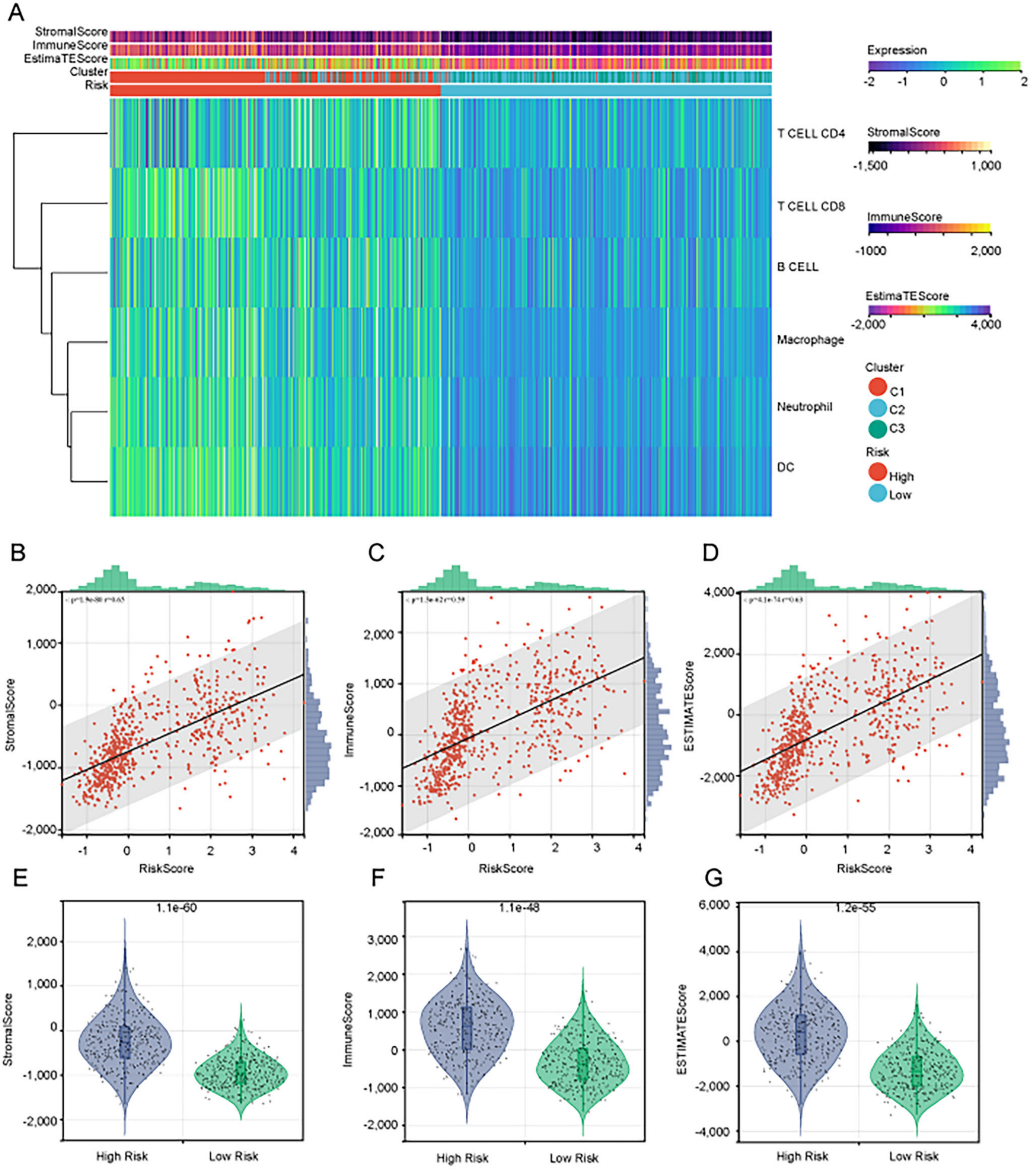
Correlation between CLOCK‐related lncRNAs and the degree of immune cell infiltration in the tumor microenvironment. (A) Heatmap depicting the immune cell infiltration scores calculated by TIMER in the high and low CLOCK‐related lncRNA risk groups for TCGA‐derived samples, (B–D) correlation between immune infiltration scores calculated by ESTIMATE and CLOCK‐related lncRNA risk scores, and (E–G) differences in immune infiltration scores between the high‐ and low‐risk groups.

TIMER analysis of immune infiltration scores in glioma samples from TCGA and their relationship with CLOCK‐related lncRNA risk scores revealed that the high‐risk group exhibited higher levels of infiltration by various immune cells (Figure 7A). ESTIMATE analysis indicated a strong positive linear correlation between CLOCK‐related lncRNA risk scores, stromal and immune scores, and overall ESTIMATE scores in each sample (Figure 7B–C). The high‐risk group scored higher in these categories (Figures 7E–G).

## 4 Discussion

The control center of the circadian rhythm, located in the superior hypothalamic nucleus, regulates the rhythms of various physiological processes (Bhadra et al. 2017). Variations in the expression of clock genes in tumors have been associated with cell proliferation, metabolism, DNA replication and repair, aging, apoptosis, DNA damage response disruption, and increased drug resistance (Kinouchi and Sassone‐Corsi 2020). Circadian clock disruption promotes tumor growth and progression by fundamentally affecting cancer cell biology (Xuan et al. 2021, Khan et al. 2019). Studies have shown that glioma tumor growth is faster in mice with Bmal1 gene‐knockout cells (Wagner et al. 2021); Per2 imbalance is involved in carcinogenesis (Wang et al. 2014); and abnormal expression of circadian clock genes, such as BMAL1, PERs, CRY‐1, and CRY‐2, can affect tumor occurrence through various pathways, including oncogenes, tumor‐suppressor genes, cell cycle, metabolic reprogramming, tumor immune escape, disruption of endocrine homeostasis, and alteration of the gut microbiota (Zhang et al. 2021).

Immune cells, such as T lymphocytes, B lymphocytes, tumor‐associated macrophages (TAMs), natural killer (NK) cells, and DCs, can have a major influence on the progression of tumors, affecting tumor growth, immune monitoring, tolerance, and evasion of the body's antitumor mechanisms (Oh et al. 2020, Liu and Guo 2018, Myers and Miller 2021). Results from counting and functional immune system studies in mammals have demonstrated a marked circadian rhythm in various immune cell populations, including NK cells, monocytes, DCs, B cells, and T cells (Scheiermann et al. 2013, Lange et al. 2010, Dimitrov et al. 2007). The biological clock is a major factor in tumor immunity, including the display and presentation of cancer cell antigens, induction and activation of effector immune cells, transportation and infiltration of tumor immunity, and elimination of cancer cells. lncRNAs are epigenetic regulators that interact with genes, mRNA, and proteins to regulate gene expression and function at various stages (Bartel 2004).

The risk scores of the nine essential genes (Figure 1) associated with the clock phenomenon, which were constructed in TCGA and CGGA databases, can be used as independent prognostic factors (Figure 2) to predict the prognosis of gliomas. Correlation analysis of the nine essential clock genes revealed 100 lncRNAs that may be associated with them, indicating that lncRNAs may interact with clock genes to regulate their expression. lncRNAs are involved in various stages of tumor formation, invasion, and progression and are also involved in the conversion of mRNA, translation, and post‐translational modification regulation in the cytoplasm (Momtazmanesh and Rezaei 2021). Generally, oncogenic lncRNAs are linked to the regulation of the cell cycle, promotion of tumor proliferation, prevention of cell apoptosis, and initiation of tumor invasion and migration (Momtazmanesh and Rezaei 2021). lncRNAs are indispensable for the emergence and development of GBM, particularly in tumor‐related immune processes (Wilkes et al. 1986). Studies have shown that lncRNA‐H3K4me3 represses the transcription and translation of the PER2 gene in mice, although the specific mechanism remains unclear (Koike et al. 2012). Additionally, lncRNA‐HULC can promote tumor development by increasing the expression of clock genes, such as CLOCK during tumor development (Cui et al. 2015).

Through LASSO analysis, we identified 31 lncRNAs connected to clock gene expression, including LOXL1‐AS1, BASP1‐AS1, HOXD‐AS2, CRNDE, and MIR4435‐2HG, which can influence lncRNA‐clock risk scores and affect immune infiltration in gliomas. Overexpression of TIAR, silencing of LOXL1‐AS1, and miR‐374b‐5p suppression work synergistically to inhibit tumor growth and vasculogenic mimicry (VM) in glioma (Yi et al. 2022). Hypoxia‐related long (HRL) noncoding RNA, known as lncRNA‐BASP1‐AS1, has been shown to considerably affect the development of glioma cells. Using HRL markers, patients with similar levels of immune‐checkpoint expression can be distinguished, which may help indicate the efficacy of immune‐checkpoint inhibitors. The HRL risk score is a valuable tool for predicting the prognosis of patients with low‐grade glioma (Xu et al. 2021). HOXD‐AS2 expression is increased during glioma infiltration and is inversely associated with prognosis (Zhang et al. 2023). CRNDE is a major factor in the risk stratification of patients with glioma, since it is abundantly expressed in glioma specimens and cell lines. Impaired CRNDE expression decreases the proliferation and invasion of glioma cells (Song et al. 2022). Previous studies have anticipated and validated the lncRNA‐CRNDE/miR‐23b‐3p/IDH1 axis related to necrosis to create a prognostic model for glioma (Chen et al. 2023), which may offer assistance in immunotherapy for gliomas. MIR4435‐2HG is overexpressed in GBM tissue (Xu et al. 2020). Studies have demonstrated that decreasing the expression of MIR4435‐2HG can impede the growth and spread of GBM cells, whereas an increase in its expression can accelerate the progression of GBM. These results suggest that MIR4435‐2HG can act as a stimulant for tumor proliferation and invasion, making it a potential marker for early tumor diagnosis and a possible target for cancer therapy (Zhao et al. 2022). The identification of lncRNAs has provided new insights into the pathological mechanisms of various diseases, including cancer (Yu et al. 2021). LOXL1‐AS1, BASP1‐AS1, HOXD‐AS2, CRNDE, and MIR4435‐2HG are lncRNAs correlated with key clock genes.

Increasing evidence suggests that abnormal lncRNAs act as new markers linked to antitumor immunoreactivity (Yu et al. 2021). lncRNAs are strongly associated with the proliferation and invasion of gliomas. Moreover, lncRNAs can control the circadian clock in gliomas. Over the last few years, immunotherapy has advanced substantially and transformed into a therapeutic modality for solid tumors (Zhang and Zhang 2020, Li et al. 2022). Some of the approaches employed in immunotherapy include vaccine therapy, immune‐checkpoint therapy, chimeric antigen receptor T‐cell (CAR‐T) immunotherapy, NK cell therapy, and oncolytic virus therapy (Sampson et al. 2020, Ettinger et al. 2022). This newly established clock‐related lncRNA expression model can be employed to identify potential targets for glioma biological clock immunotherapy and to gauge the prognosis of patients with GBM.

Although the analysis results were reliable, research based on public databases still shows some limitations. Cell and tissue experiments are essential to gain a better understanding of the molecular mechanisms by which lncRNAs regulate the function of clock genes. Without performing algorithm benchmark testing or hyperparameter optimization, LASSO was chosen as the machine learning algorithm; however, it may not be the most suitable algorithm for the lncRNA‐clock risk score. Through transcriptome and genomic analyses, this study yielded valuable insights; however, the information obtained at the batch level tended to be aggregated across multiple cells, which can often hide particular information. To gain a better understanding of the potential role of lncRNAs in immunotherapy for glioma biological clocks, studies with larger sample sizes and in vivo models are essential.

## Author Contributions

The principal investigators: Mingjie Gongand Chengfa Sun. Data design: Zhenhua Shi. Data analysis: Junxiang Wang. Drafting of the manuscript: Weiwei Zhai and Zhengquan Yu. All the authors have approved the final version for publication.

## Conflicts of Interest

The authors declare no conflicts of interest

## Ethics Statement

All samples included in this study were obtained from the online databases Cancer Genome Atlas (TCGA), Chinese Glioma Genome Atlas (CGGA), and Genotype‐Tissue Expression (GTEx) without the need for ethical review.

## Peer Review

The peer review history for this article is available at https://publons.com/publon/10.1002/brb3.71000.

## Supporting information




**Supplementary Table 1** Primer sequences required for the qPCR of the four lncRNAs

**Supplementary Table 2** mRNAs expression differences between the clusters

**Supplementary Table 3** lncRNA expression differences between the clusters

**Supplementary Table 4** Intersection results of CLOCK‐related lncRNA

**Supplementary Table 5** Clock‐related lncRNAs after LASSO‐Cox filtering

## Data Availability

All data generated or analyzed during this study are included in this published article and its supplementary information files.
