# Supplementary material for: Constructing a Model Using Clock‐Related lncRNAs for Predicting the Tumor Microenvironment of Gliomas
Source: Brain Behav. 2025 Oct 15;15(10):e71000. doi: 10.1002/brb3.71000 (PMC12528804; doi:10.1002/brb3.71000)
Supplement: Supplementary file 1 — Supplementary Table 1 Primer sequences required for the qPCR of the four lncRNAs [file BRB3-15-e71000-s005.docx]

**Supplementary Table 1 Primer sequences needed for the qPCR of the four lncRNAs**

| **lncRNA ID** | **Primer sequences (5’-3’)** |
| --- | --- |
| ENSG00000230404 F1 | CATCGGAATCTCACAACCACC |
| ENSG00000230404 R1 | GGCTTTGCCACCTACCATCT |
| ENSG00000236106 F1 | AAAGTCATTCACTGCTGCCTCT |
| ENSG00000236106 R1 | CAGAAGATTACGGTTGGATCAACT |
| ENSG00000249304 F1 | ACACTTCTTGCTCTCCTGCGA |
| ENSG00000249304 R1 | GGGAATCCAAACTGCTTCCT |
| ENSG00000269416 F1 | TGGGATCGCTTTCTGCTATTCA |
| ENSG00000269416 R1 | TAGCTGTCTGTGCACGTCCC |
